# Spatio-temporal model of Meox1 expression control involvement of Sca-1-positive stem cells in neointima formation through the synergistic effect of Rho/CDC42 and SDF-1α/CXCR4

**DOI:** 10.1186/s13287-021-02466-8

**Published:** 2021-07-07

**Authors:** Yan Wu, Yuan-jin Li, Liu-liu Shi, Yun Liu, Yan Wang, Xin Bao, Wei Xu, Lu-yuan Yao, Magdaleena Naemi Mbadhi, Long Chen, Shan Li, Xing-yuan Li, Zhi-feng Zhang, Sen Zhao, Ruo-nan Zhang, Shi-You Chen, Jing-xuan Zhang, Jun-mingTang

**Affiliations:** 1 Department of Physiology, Hubei Key Laboratory of Embryonic Stem Cell Research, Faculty of Basic Medical Sciences, Hubei University of Medicine, Shiyan, 442000 Hubei People’s Republic of China; 2 Hebei Medical University, Shijiazhuang, 050017 Hebei People’s Republic of China; 3 Cental Lab, Guoyao-Dongfeng Hospital, Hubei University of Medicine, Shiyan, 442000 Hubei People’s Republic of China; 4 Department of Biochemistry, Faculty of Basic Medical Sciences, Hubei University of Medicine, Shiyan, 442000 Hubei People’s Republic of China; 5 Faculty of Basic Medical Sciences, Institute of Biomedicine, Hubei University of Medicine, Shiyan, 442000 Hubei People’s Republic of China; 6 The Department of Surgery, University of Missouri, Columbia, USA

**Keywords:** Meox1, Sca-1, Neointima, SDF-1α, CDC42

## Abstract

**Aims:**

Neointimal hyperplasia remains a major obstacle in vascular regeneration. Sca-1-positive progenitor cells residing within the vascular adventitia play a crucial role in the assemblage of vascular smooth muscle cell (VSMC) and the formation of the intimal lesion. However, the underlying mechanisms during vascular injury are still unknown.

**Methods and results:**

Aneointimal formation rat model was prepared by carotid artery injury using 2F-Forgaty. After vascular injury, Meox1 expressions time-dependently increased during the neointima formation, with its levels concurrently increasing in the adventitia, media, and neointima. Meox1 was highly expressed in the adventitia on the first day after vascular injury compared to the expression levels in the media. Conversely, by the 14th day post-injury, Meox1 was extensively expressed more in the media and neointima than the adventitia. Analogous to the change of Meox1 in injured artery, Sca-1+ progenitor cells increased in the adventitia wall in a time-dependent manner and reached peak levels on the 7th day after injury. More importantly, this effect was abolished by Meox1 knockdown with shRNA. The enhanced expression of SDF-1α after vascular injury was associated with the markedly enhanced expression levels of Sca1+ progenitor cell, and these levels were relatively synchronously increased within neointima by the 7th day after vascular injury. These special effects were abolished by the knockdown of Meox1 with shRNA and inhibition of CXCR4 by its inhibitor, AMD3100. Finally, Meox1 concurrently regulated SDF-1α expressions in VSMC via activating CDC42, and CDC42 inhibition abolished these effects by its inhibitor, ZCL278. Also, Meox1 was involved in activation of the CXCR4 expression of Sca-1+ progenitor cells by CDC42.

**Conclusions:**

Spatio-temporal model of Meox1 expression regulates theSca-1+progenitor cell migration during the formation of the neointima through the synergistic effect of Rho/CDC42 and SDF-1α/CXCR4.

**Supplementary Information:**

The online version contains supplementary material available at 10.1186/s13287-021-02466-8.

## Introduction

Coronary heart diseases are the most prevalent diseases worldwide and the leading cause of death worldwide. Surgical intervention with percutaneous coronary intervention (PCI) is the most effective therapy for coronary heart disease. However, the early treatments with balloon angioplasty (BA) and bare-metal stents (BMS) accompanied increased risk of restenosis [1, 2]. The introduction of drug-eluting stents (DES) proved to have significant improvement in reducing in-stent restenosis (ISR) incidents and thus have become the standard use with PCI therapy [1]. Although DES has shown superior in preventing ISR, introducing a stent inhibits the proliferation of vascular endothelial cells and smooth muscle cells (SMC) in neointima, resulting in re-endothelialization, which ultimately leads to late stent thrombosis (LST) or very-LST (VLST). This warrants the need for prolonged use of dual anti-platelet therapy (DAPT), which increases the risk of bleeding and further is an economic burden for patients [2–4]. Therefore, it is urgent to study and understand the underlying mechanisms that drive the pathology restenosis after surgical intervention and explore new target therapy to reduce the physical and economic burden for patients.

SMC are the primary cells involved in the formation of the neointima. Although it has been well accepted that the SMC that participate in the neointima lesion originate from the vascular media [2–5], both bone marrow-derived and adventitia derived progenitor cells can be activated and migrate to the intima where they differentiate into SMCs and participate in the neointima formation [6–8]. Further, based on stem cell genetic tracking technology, Sca-1+ progenitor cells are in the inner wall of the adventitia and not from the bone marrow [9–12]. It is, therefore, believed that the mobilization and recruitment of Sca-1+ progenitor cells from the adventitia and media into the intimal wall is the main mechanism that leads to the accumulation of SMCs in the neointima during vascular wall remodeling, such as intimal hyperplasia and arteriosclerosis [6, 7, 9, 13–17]. However, the mechanisms involving the spatio-temporal distribution of Sca-1+ progenitor cells during the process of neointima formation are still unknown.

Homeobox gene (HOX) is a family of transcription factor genes that are essential for cell differentiation. They include the mesoderm/mesenchymal homeobox gene 1 (MEOX1) and MEOX2 that have regulatory roles during cell proliferation, differentiation, and migration [18–20]. Some studies have found that MEOX1 expression was enhanced after vascular balloon injury-induced in the rat model, accompanied by SMCs proliferation, migration, and increased neointima formation. Knockdown of MEOX1 significantly inhibited the proliferation and migration of SMCs, leading to the inhibition of neointima formation [21, 22]. This suggests that the formation of the neointima is related to the proliferation and migration of SMCs mediated by MEOX1.

In this study, we found that MEOX1 expression gradually increased in a time-dependent manner with the gradual increase of neointima formation in injured arteries and expressed traits of increased expression in the adventitia, media and neointima. Importantly, change in the expression of MEOX1 in the injured artery was associated with the gradual increase of Sca-1+ progenitor cells in the adventitia wall in a time-dependent manner. Thus, the spatio-temporal distribution pattern of Sca-1+ progenitor cells in vascular injury is closely related to the spatio-temporal pattern of MEOX1 expression.

## Method

### Animal

According to the Guide of China Laboratory Animals for Care and Use, all animals were raised in a specific-pathogen-free (SPF) grade animal lab. All the procedures involving the animals were approved by the Committee of Experimental Animals Care of Hubei University of Medicine.

### Human samples

The study was performed strictly following ethical guidelines for biomedical research involving human subjects in China and was approved by the Institutional Review Board of Shiyan Renmin Hospital, Hubei University of Medicine. Written informed consent was obtained from all participating individuals. Human umbilical cords were collected before disposal after babies were born in Shiyan Renmin Hospital.

### Human umbilical cord Sca-1+ progenitor cells isolation and culture

Human umbilical cord Sca-1+ progenitor cells were isolated according to the previously described protocol [16]. Briefly, the media layer of the human umbilical cord was carefully removed. The adventitial tissues were collected and cut into 0.5-mm pieces and cultured on 10-cm plates in a 5% CO_2_ incubator at 37 °C for 3 h before adding the stem cell growth medium. After 5~7 days of incubation, the migrated cells from the adventitial tissues were digested with 0.25% pancreatic enzymes and collected for purification. The purity of selected Sca-1^+^ progenitor cells was evaluated by immunofluorescence staining of Sca-1+ progenitor cells.

### VSMC culture

Primary vascular smooth muscle cells (VSMC) were isolated from aortic artery of Sprague Dawley rats (280–300 g) and cultured as previously described [13]. Upon reaching 60% cell confluence, the cells were transfected with Ad-Null, Ad-MEOX1, or Ad-shMEOX1 for 24 h. The culture media were changed after that.

To detect the relationship between MEOX1 and SDF-1α, the cells were transfected with Ad-MEOX1 at different multiplication of infection (MOI) for 3 days.

For the inhibition experiment, VSMC were transfected with Ad-Null or Ad-MEOX1 or Ad-shMEOX1 followed by treatment with a vehicle (CTL) or selective inhibitors of RhoA (CCG1423,10 μM), CDC42 (ZCL278,10 μM), or Rac1 (Azathioprine1 μM).

To observe the effects of Meox1 on Sca-1+ progenitor cell migration, conditioned media (CM) containing VSMC were prepared. Briefly, the corresponding CM of VSMC was collected 3 days after transfected with Ad-Null (CM-Ctrl) and Ad-Meox1 (CM-Meox1) as previously described [13].

### Carotid artery balloon injury model and adenoviral gene transfer

Carotid artery balloon injury model was performed as described previously [23] with 2F-Forgaty catheter (CA92614-5686, Edwards Life sciences LLC Co.). The artery segment from the proximal edge of the omohyoid muscle to the carotid bifurcation was exposed and washed with saline and incubated with 100 μl of saline or adenovirus expressing Meox1 shRNA (5 × 10^9^ pfu) or AMD3100 (10 μM) via a fixed catheter for 20 min [16, 21, 24]. Seven or 14 days later, the rats were euthanized with isoflurane. Then, the balloon-injured and adenovirus-dwelled segment was perfused with saline and collected for follow-up detection and analysis.

### Grouping of animal experiments

To confirm the relationship among spatio-temporal characteristics of Meox1, Sca-1+ cells, and SDF-1α expression, rats were divided into the following groups: after balloon injury, arteries samples 6 animals each group were collected at 0, 1, 3, 7, and 14 days after operation for detection and analysis.

To observe the relationship among Meox1, Sca-1+ cell migration, and neointimal formation, the rats were divided into sham operation group, balloon injury plus Ad-Null treatment group, and balloon injury plus Ad-shMeox1 treatment group, 6 rats each group.

To observe the correlation between Meox1-mediated Sca-1 migration and SDF-1α, the rats were divided into sham operation group, balloon injury plus vehicles group, and balloon injury plus AMD3100 group, 6 rats each group.

### Histomorphometric analyses

Common carotid artery segments were prepared at a thickness of 5 μm and stained with hematoxylin and eosin (H&E) for morphometric analyses. The images of 8-view fields of each cross-section were randomly taken under a Nikon microscope (Nikon America, Inc.). Using special software (Image-Pro Plus, Media Cybernetics), the lumen, internal elastic lamina, and external elastic lamina areas were determined by two double-blind pathologists. The ratio of intimal and medial area (I/M) was assessed with the following formula: I/M ratio (%) = [IEL area − lumen area]/[EEL area − IEL area] × 100 (%) [13].

### Immunohistochemistry and immunofluorescent staining

After rehydration and antigen retrieval, artery sections at different time points were blocked with 5% goat serum and permeabilized with 0.01% Triton X-100 in PBS, and incubated with Meox1 antibody at 4 °C overnight followed by incubation with HRP-conjugated secondary antibody for immunochemistry staining. The sections were counterstained with hematoxylin.

To observe theSca-1+ progenitor cell migration from the adventitia into the intima, the artery sections at different time points were incubated with Sca-1 (1:200, ab25031, Abcam) antibody followed by fluorescent dye-conjugated secondary antibody (Jackson Immuno Research) and counterstained with DAPI (Sigma) [25].

To detect SDF-1α expression in VSMC of the injured vessels, we used co-immunofluorescent staining of artery sections at different time points and incubated with α-SMA (1:150, sc-130616, Santa Cruz) and SDF-1α (1:150, ab9797, Abcam) antibodies followed by fluorescent dye-conjugated secondary antibody (Jackson ImmunoResearch) and counterstained with DAPI (Sigma).

To observe the intermediate state of double-positive Sca1+ progenitor cells and VSMCs, we used co-immunofluorescent staining of Sca-1 (1:200, ab25031, Abcam) and α-SMA (1:150, sc-130616, Santa Cruz). The artery sections were incubated 7 or 14 days after treatment with Ad-shMeox1 or AMD 3100, and the corresponding fluorescent dye-conjugated secondary antibody (Jackson ImmunoResearch) were used and counterstained with DAPI (Sigma).

For semi-quantitative analysis, fluorescence values or gray values of images (three random fields each artery section, n = 6) were analyzed by using Image-Pro Plus 6.0.

### Western blotting

Protein was extracted using RIPA lysis buffer containing protease inhibitor mix. Protein concentration was measured using BCA Protein Assay Kit. Twenty micrograms protein samples were separated on 4–12% SDS-polyacrylamide gels, and electro-transferred onto PVDF membranes (Bio-Rad). The membranes were incubated at 4 °C overnight against antibodies SDF-1α (ab9797, 1:500, Abcam), CXCR4 (1:500), or α-Tubulin (1:5000, T6074, Sigma) in blocking buffer containing 5% milk followed by incubation with HRP-conjugated secondary antibody (Sigma).

### Transwell invasion assays

To determine Meox1 and SDF-1α function in regulating Sca-1+ progenitor stem cell migration, CM-Ctrl or CM-Ad-Meox1 were used to explore the cell migration under transwell system with or without ZCL278, CCG-1423, Azathioprine, or AMD3100. After 48 h of incubation, cells were fixed with 100% methanol and stained with crystal violet (0.1%). The cells in the upper membranes were removed, and the remaining cells at the bottom of the membrane were imaged under a light microscope and mounted for quantitative analysis. Images of 3 different view fields in each transwell membrane were acquired with an optical microscope using a 10× magnification. Each of the 3 independent experiments was repeated in triplicates. Migration index was assessed as follows: treatment group cells number/control group cells number.

### Statistical analysis

All data were expressed as mean ± SD and then evaluated with a 2-tailed, unpaired Student t-test or compared by one-way ANOVA followed by the t-test. A value of *P* < 0.05 was considered statistically significant.

## Results

### Meox1 and Sca-1+ stem cells are induced during neointimal formation and express spatio-temporal traits during balloon injury-induced artery

**Fig. 1:**
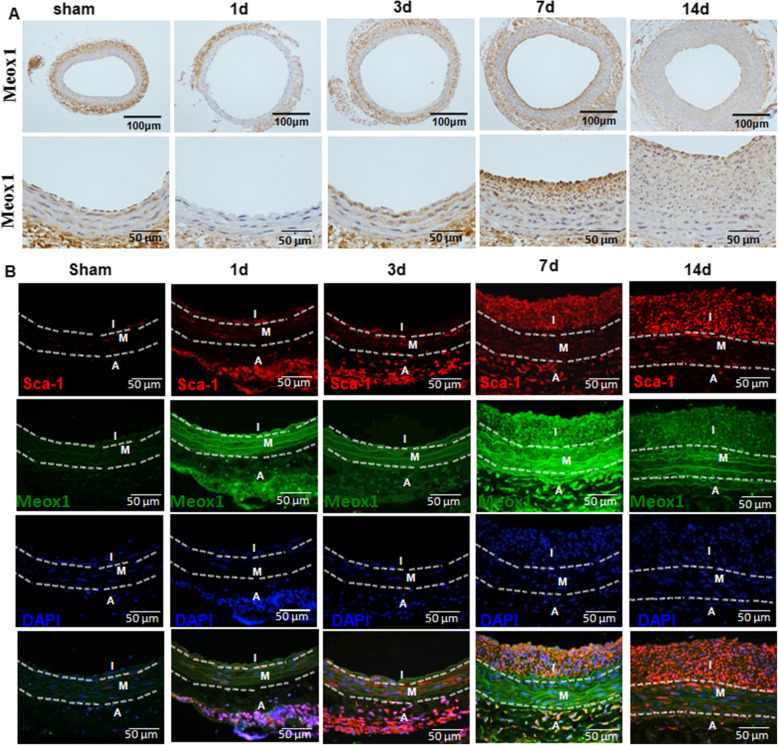
Traits spatio-temporal expression of Meox1 and Sca-1+ stem cells during balloon injury-induced neointimal formation. The experiment preliminarily demonstrated the traits of distribution of both Sca-1+ progenitor cells and Meox1 expression in the vessel wall during the process of neointimal formation after vascular injury and identified the correlation between them. **A** Rats underwent carotid artery balloon injury. Representative sections of sham operation and injured arteries at the indicated time points were stained with Meox1. Open arrow indicates the neointima area, forked tail arrow indicates the media, and round arrow indicates the adventitia. **B** Immunofluorescence staining of Sca-1+ and Meox1+ cells on the first and fourteenth day after injury. Red fluorescence indicates Sca-1; green indicates Meox1; blue fluorescence indicates DAPI-labeled nucleus; I, neointima; M, media; A, adventitia

To identify the expression of Meox1 and Sca-1+ progenitor cells during neointimal formation, we detected the expressions of Sca-1+ and Meox1 at day 1, day 3, day 7, and day 14 after balloon-injured carotid artery. Immunohistochemical analysis revealed that Meox1 gradually increased in a time-dependent manner from the adventitia to media and into the neointimal (Fig. 1A, B). This was accompanied by the progressive increase of neointimal formation after carotid artery injury. Compared with the sham group, Meox1 expression was significantly increased on day 1 after injury. Meanwhile, within the injured artery, Meox1 expression showed greater levels in the adventitia wall than the media on day 1 after injury (Fig. 1 and sFigure1). In contrast, the Meox1 levels were significantly lower in the adventitia wall than in the media or neointimal on day 14 after injury.

Concurrently, Sca-1+ progenitor cells were also detected in the adventitia wall on the first day after vessel injury and gradually increased in a time-dependent manner, reaching significant levels on day 7 in the intima. Notably, Sca-1+ progenitor cells were not detected within the intimal wall at least in the first 3 days post-injury. However, they were markedly increased by day 7 post-injury, reaching apex levels on day 14 after injury (Fig. 1, and Additional file. FigureS1). Similarly to the Meox1 expression trait, Sca-1+ progenitor cells show increased levels from the adventitia to the media and the intimal wall in a time-dependent manner.

These results suggest that the expression of Sca-1+ progenitor cells in the vessel wall was associated with Meox1 expression.

### Meox1 promotes the migration of Sca-1+positive stem cells into the neointima

**Fig. 2:**
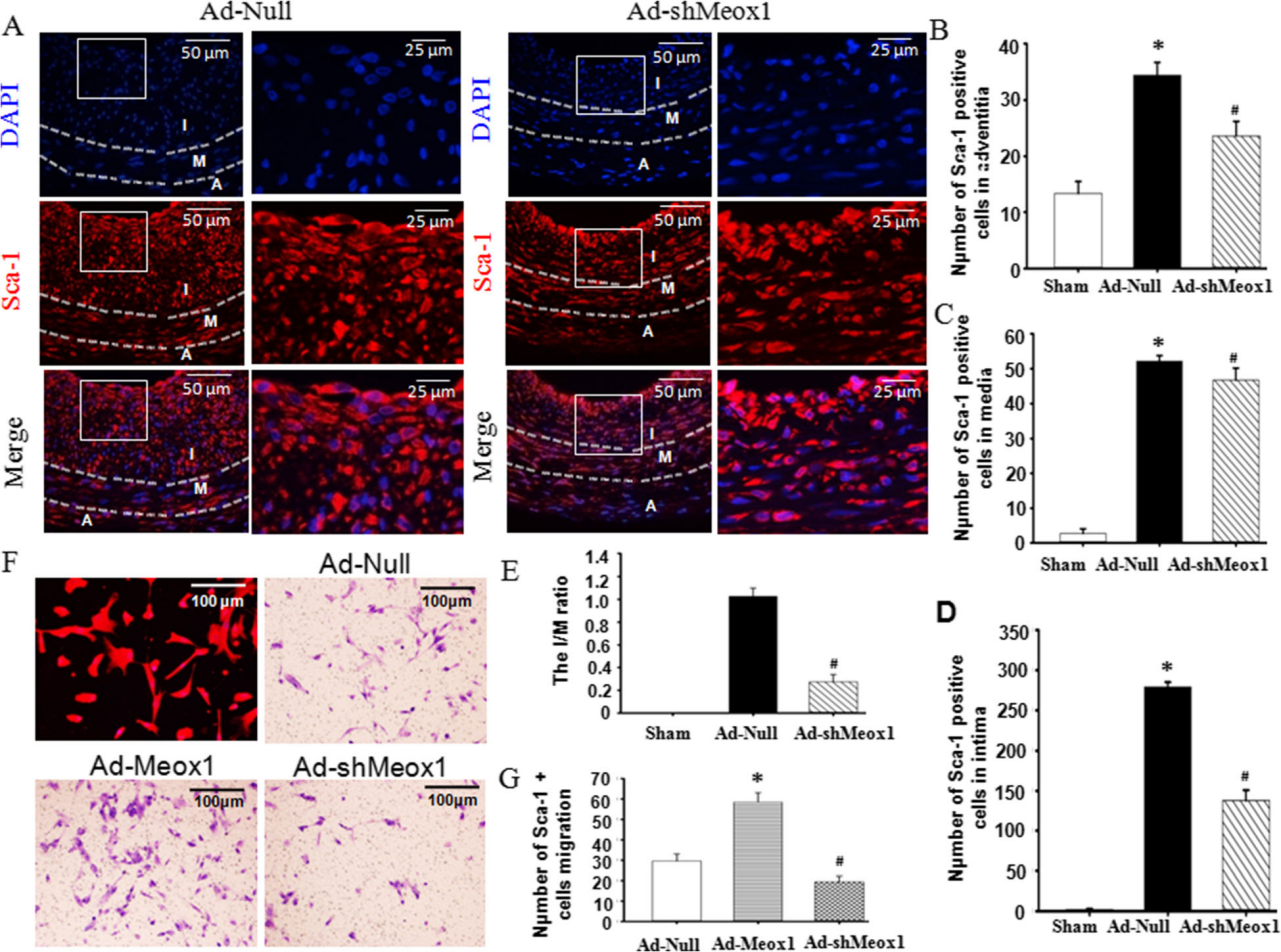
Meox1 promoted Sca-1-positive stem cell migration into neointima. **A** Fluorescence staining for Sca-1 in injured arteries treated with or without Ad-shMeox1 for 14 days. Round arrow indicates the neointima area, open arrow indicates the media, and forked tail arrow indicates the adventitia. **B**–**D** The number of Sca-1+ progenitor cells were determined within the media or intima of **A** by Image-Pro Plus software. Six rats each group, random three high power visual field each section, n = 6, ^&^*P* < 0.05 vs. the injured arteries treated with Ad-Null. **E** The I/M ratio from **A**, *P < 0.05 vs. Ad-Null. **F** Response of human umbilical cord Sca-1 progenitor cell migration to conditioned medium (CM) of VSMC treated with or without Ad-Meox1. **G** Quantitative analysis of cell migration was analyzed using the Transwell system. n = 15, *P < 0.05 vs. CM-Ctrl; ^#^*P* < 0.05 vs. CM-Ad-Meox1. I, neointima; M, media; A, adventitia

Considering the expression traits observed in Meox1 and Sca-1+ positive stem cell during the formation of the neointima, we treated balloon-injured carotid arteries with Ad-shMoex1. Using immunofluorescence staining, we knockdown Meox1 and assessed the expression of Sca-1+_ progenitor cells in the media and neointimal. As shown in Fig. 2A–D, the numbers of Sca-1+ progenitor cells in Ad-shMeox1-treated arteries were drastically decreased within the media wall and the neointimal lesion compared with Ad-Null-treated arteries. This indicates that Meox1 participated in Sca-1+ progenitor cell migration into the neointimal. Furthermore, the I/M ratio in the Ad-shMeox1-treated arteries was lower than in the Ad-Null-treated arteries (Fig. 2E). In vitro, the overexpression of Meox1 promoted Sca-1 progenitor cell migration. By contrast, knockdown of Meox1 by shRNA inhibited Sca-1 progenitor cell migration (Fig. 2F, G). These results suggest that Meox1 is involved in neointima formation after vascular injury, likely via inducing Sca-1+ progenitor cell migration into neointima.

### Meox1 promoted Sca-1^+^ stem cell migration into neointima through activating Rho/CDC42 signaling

**Fig. 3:**
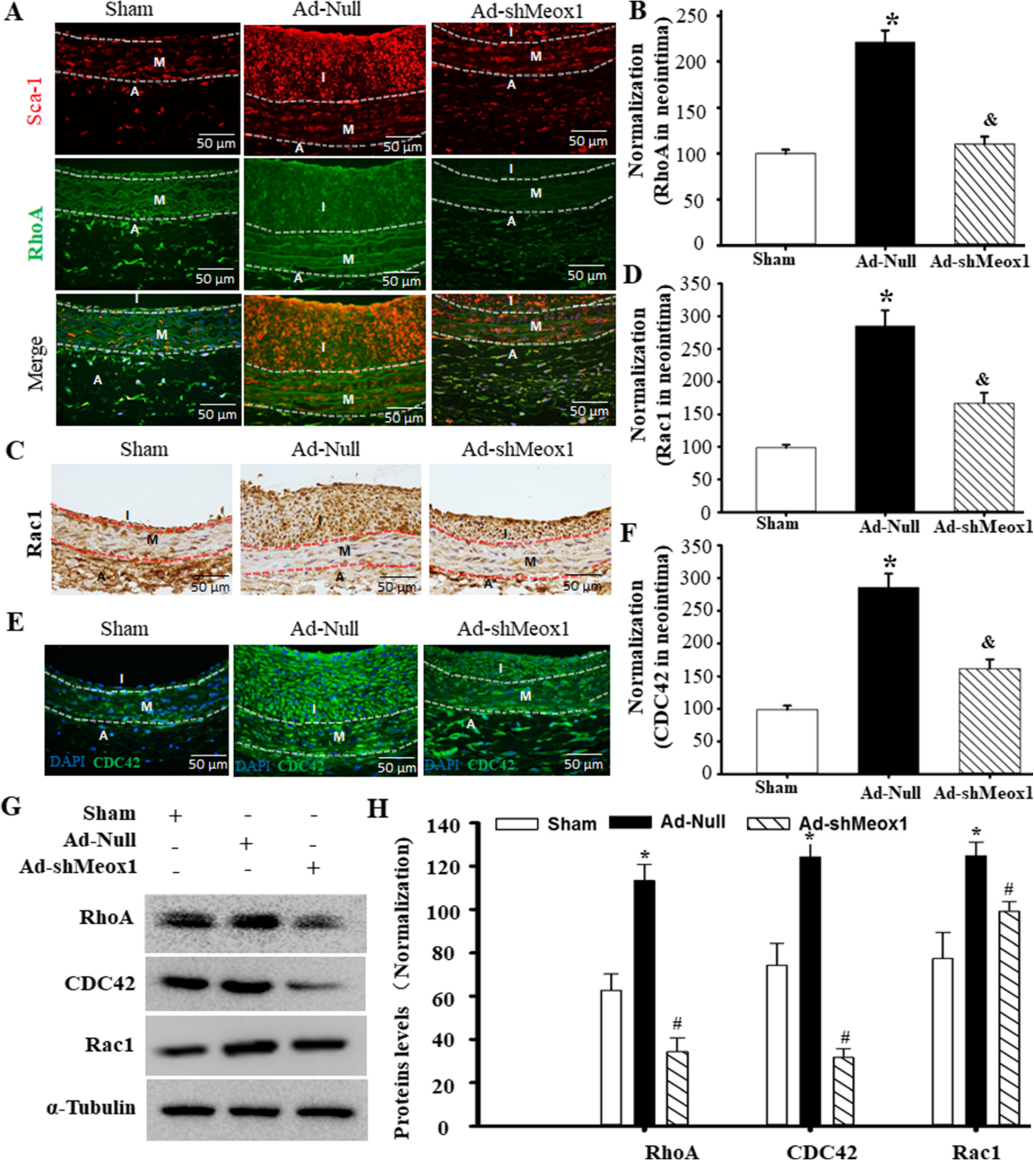
Meox1 promoted Sca-1^+^ stem cell migration into neointima through activating Rho/CDC42 signaling. **A** Immunofluorescence (IF) double staining for Sca-1 and RhoA in injury arteries treated with or without Ad-shMeox1 for 14 days. Red fluorescence indicates Sca-1, green fluorescence indicates RhoA, and blue fluorescence indicates DAPI-labeled nucleus. I, neointima; M, media; A, adventitia. **B** RhoA expressions in neointima were determined within figure 3A by Image-Pro Plus software. n = 6, *P < 0.05 vs. sham; ^&^*P* < 0.05 vs. the injured arteries treated with Ad-Null. **C**–**D** Rac1 expression in neointima were detected by IF staining (**C**) and analyzed by Image-Pro Plus software. Six rats each group, random three high power visual field each section, n = 6, *P < 0.05 vs. sham; ^&^*P* < 0.05 vs. the injured arteries treated with Ad-Null. **E** CDC42 expression by IF staining; **F** CDC42 expressions in neointima were determined within **E** by Image-Pro Plus software. n = 18, *P < 0.05 vs. sham; ^&^*P* < 0.05 vs. the injured arteries treated with Ad-Null. **G**, **H** Knockdown of Meox1 by shRNA reduced proteins levels of RhoA, Rac1, and CDC42 in the injured arteries transfected with treatment of Ad-shMeox1 for 14 days as determined by western blot (**G**) and semi-quantitative analysis (**H**). n = 3, *P < 0.05 vs. sham; ^#^*P* < 0.05 vs. Ad-Null. I, neointima; M, media; A, adventitia

RhoA and Rac-1 are two mutual antagonists’ members of the Rho family that play a crucial role during cell migration by regulating cell retraction or protrusion [26, 27]. CDC42 is also a member of the small Rho-family GTPase that is a central regulator of cell polarity. We explored whether Meox1 promotion of Sca-1+ progenitor cell migration into the neointima involved the small Rho-family GTPases. Using double immunofluorescence staining of Sca-1+ progenitor cells and RhoA in the artery, we found that RhoA was co-expressed with Sca-1+ progenitor cells within the adventitia, media, and neointima (Fig. 3A, B). Importantly, we observed that both Rac1 and CDC42 were significantly expressed within the neointima lesion and downregulated in Ad-shMeox1-treated arteries (Fig. 3C–F). Additionally, western blot assay showed similar changes in RhoA, Rac-1, and CDC42 in injured arteries, and these changes could be obviously abolished by knockdown of Meox1 (Fig. 3G, H). These results illustrate that the Sca-1+ progenitor cell migration into neointima may likely involve Rho/CDC42 signaling.

### Meox1 induce Sca-1+ stem cell migration into neointima through activating SDF-1 α signaling in α-SMA+ cells

**Fig. 4:**
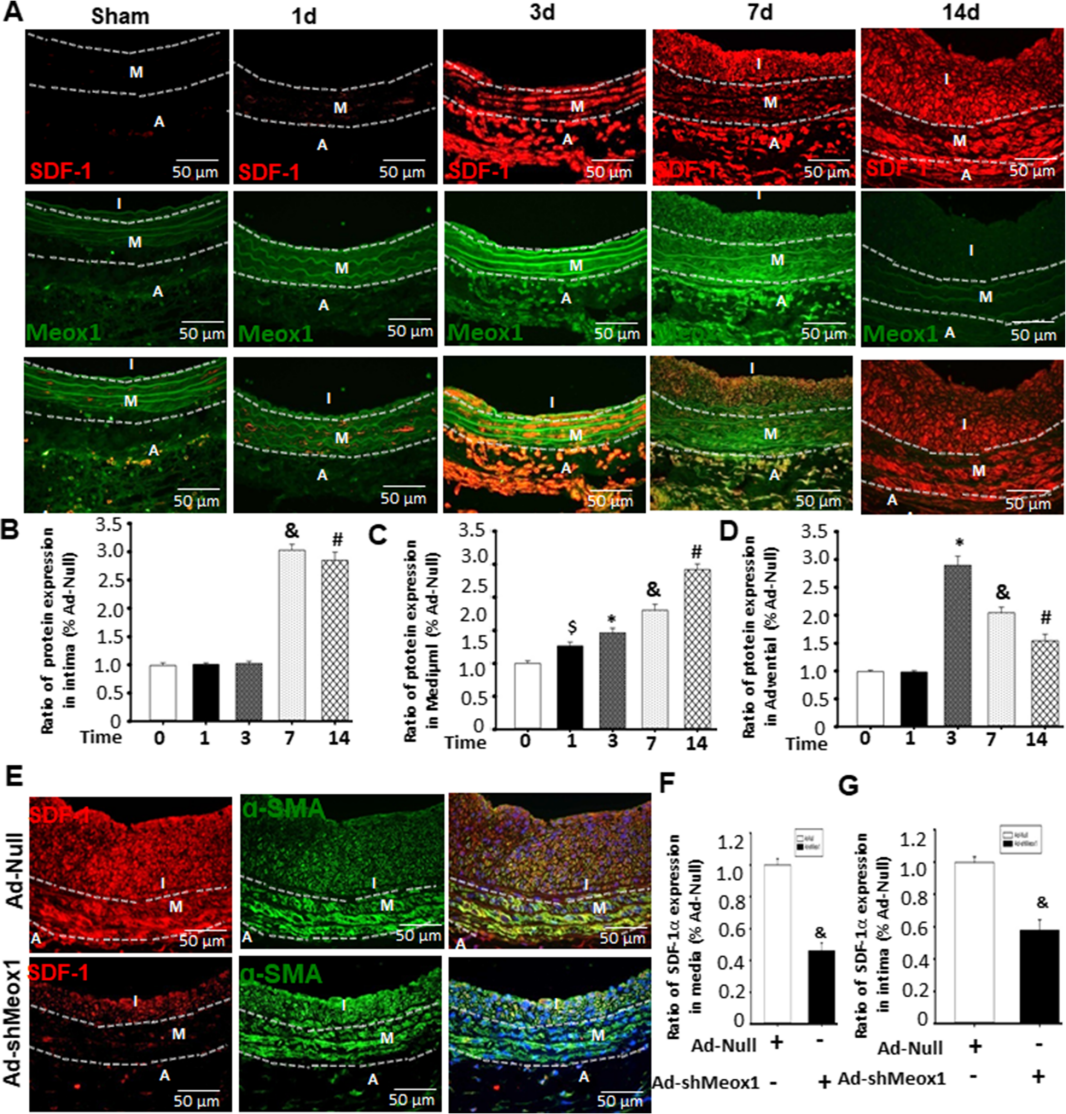
Meox1 induce Sca-1^+^ stem cell migration into neointima through activating SDF-1 α signaling in α-SMA+ cells. On the basis of results of Fig. 1, the experiment preliminary demonstrated the order of expressions of Meox1 and SDF-1α in the process of neointimal formation after vessel injury, tried to identify the correlation between expressions of Meox1 and SDF-1α. **A** Immunofluorescence double staining for SDF-1α and Meox1 in injury arteries in the indicated time. Red fluorescence indicates SDF-1α, green fluorescence indicates Meox1, and blue fluorescence indicates DAPI-labeled nucleus. Round arrow indicates lumen side neointima, open arrow indicates media, and forked tail arrow indicates adventitia. **B**–**D** Semi-quantitative analysis of optical density value of immunohistochemical staining of SDF-1α in **A** were determined within media or intima of sham operation and injury arteries by Image-Pro Plus software; percentage of SDF-1α expression at media or intima in the indicated time compared to sham group were calculated, showing the traits of time-dependent manner. n = 6, ^$^*P* < 0.05 vs. 0 day; *P < 0.05 vs. 1 day; ^&^*P* < 0.05 vs. 3 days; ^#^*P* < 0.05 vs. 7 days. **E** SDF-1α expression in injury arteries treated with Ad-shMeox1 for 14 days. **F**, **G** Semi-quantitative analysis ofSDF-1α in VSMC in injury arteries of **E**. n = 3, ^&^*P* < 0.05 vs. Ad-Null. I, neointima; M, media; A, adventitia

SDF-1 α has a functional role in homing and maintaining stem and progenitor cells to injured sites [28, 29]. We, therefore, explored whether the activation of Sca-1+ progenitor cell migration into the neointima by Meox1 involved SDF-1α/CXCR4. As shown in Fig. 4A–D, similarly to Meox1 expression traits in the injured artery, SDF-1α expressions gradually increased in the adventitia wall in a time-dependent manner, reaching apex levels on the third day after injury. In the vascular media wall, SDF-1α expressions gradually increased from day 1 post-injury and reached peak levels on day 14 post-injury. Of note, SDF-1α was not expressed in the intimal or neointimal during the early phase of vascular injury. However, it was expressed at least 3 days after injury with peak levels on day 7 post-injury (Fig. 4A–D, sFigure2A). Furthermore, these above effects could be markedly abolished by knockdown of Meox1 by shRNA. Additional, western blot showed that knockdown of Meox1 by shRNA reduced SDF-1α protein levels in injured arteries (sFigure2B-2C). To further confirm the relationship between Meox1 and SDF-1α, different MOI Ad-Meox1 were used to treat VSMC in vitro, as shown in sFigure2D-2E, Meox1 dose-dependently induced SDF-1α expression in VSMC. By combining these results of Meox1 and Sca-1+ progenitor cells in Figs. 1 and 2, we suggest that SDF-1α participates in the process of Meox1-mediated Sca-1+ progenitor cell migration into neointimal after vascular injury.

### Meox1 triggered Sca-1-positive stem cell migration into neointimal through CXCR4

**Fig. 5:**
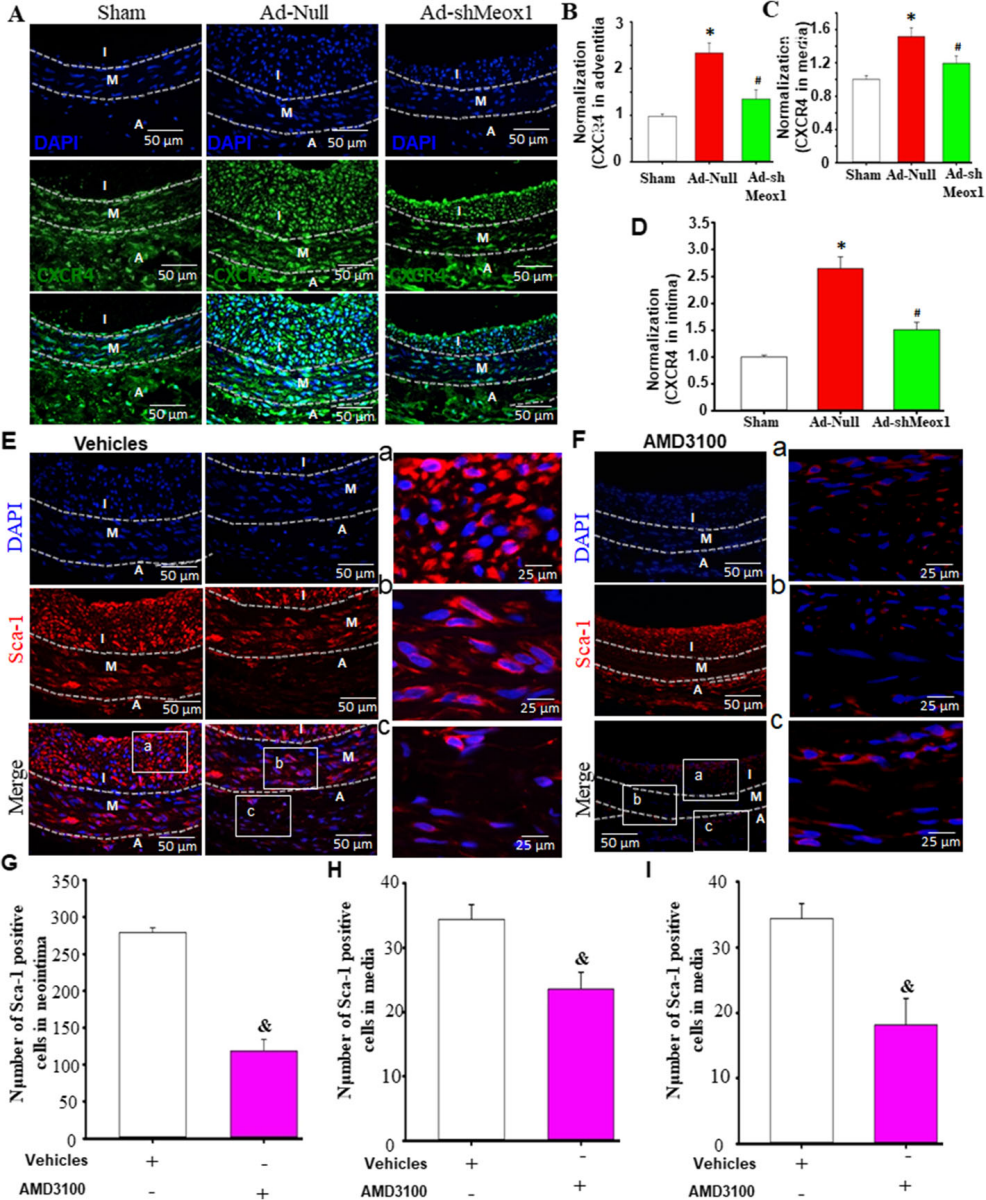
Meox1 triggered Sca-1-positive stem cell migration into neointimal through CXCR4. The experiment was designed to determine if the increased SDF-1α in injured vessels induced Sca-1-positive progenitor cell migration into neointima, using SDF-1α receptor CXCR4 blocker AMD3100 to affirm the role of SDF-1α on Sca-1-positive progenitor cell migration 7 days after vessel injury with or without AMD3100 application in vivo. **A** Immunofluorescence staining for CXCR4 in injury arteries for 7 days. Green fluorescence indicates CXCR4; blue fluorescence indicates DAPI-labeled nucleus. **B**–**D** Typical image and semi-quantitative analysis of double positive with CXCR4 were determined within media or intima of injured arteries treated with Ad-shMeox1 for 14 days by Image-Pro Plus software. Green fluorescence indicates CXCR4; blue fluorescence indicates DAPI-labeled nucleus. n = 6, *P < 0.05 vs. sham; ^#^*P* < 0.05 vs. the injured arteries treated with Ad-Null. **E**, **F** Immunofluorescence staining of Sca-1 progenitor cells in neointima of injured arteries treated with AMD3100. Red fluorescence indicates Sca-1; blue fluorescence indicates DAPI-labeled nucleus. **G**–**I** AMD3100 decreased number of Sca-1-positive progenitor cells in neointima of **E** and **F**. n = 6, ^&^*P* < 0.05 vs. the injured arteries treated with vehicles. I, neointima; M, media; A, adventitia

Along with its ligand, SDF-1α, CXCR4 is crucial in regulating cell proliferation and migration [23]. We next examined the involvement of CXCR4 in the Meox1-Sca-1+ progenitor cell-induced neointima formation. Immunohistochemistry results from the injured artery showed that CXCR4 was significantly expressed in all three layers of the vascular wall after vascular injury, and this expression was significantly decreased in Ad-shMeox1 treated arteries (Fig. 5A–D). Next, the injured arteries were treated with a CXCR4 inhibitor, AMD3100, and immunofluorescence staining against the Sca-1+ progenitor cells antibody was performed. The results showed that knockdown of CXCR4 led to a significant decrease of infiltrating Sca-1+ progenitor cells in the media wall and neointimal layer compared to the untreated injured arteries (Fig. 5E–I). These observations confirm that Meox1 mediation of Sca-1_ progenitor cell migration into the neointima involves SDF-1α/CXCR4.

### Meox1 triggered Sca-1-positive stem cell migration through RhoA-CDC42-CXCR4 signaling

**Fig. 6:**
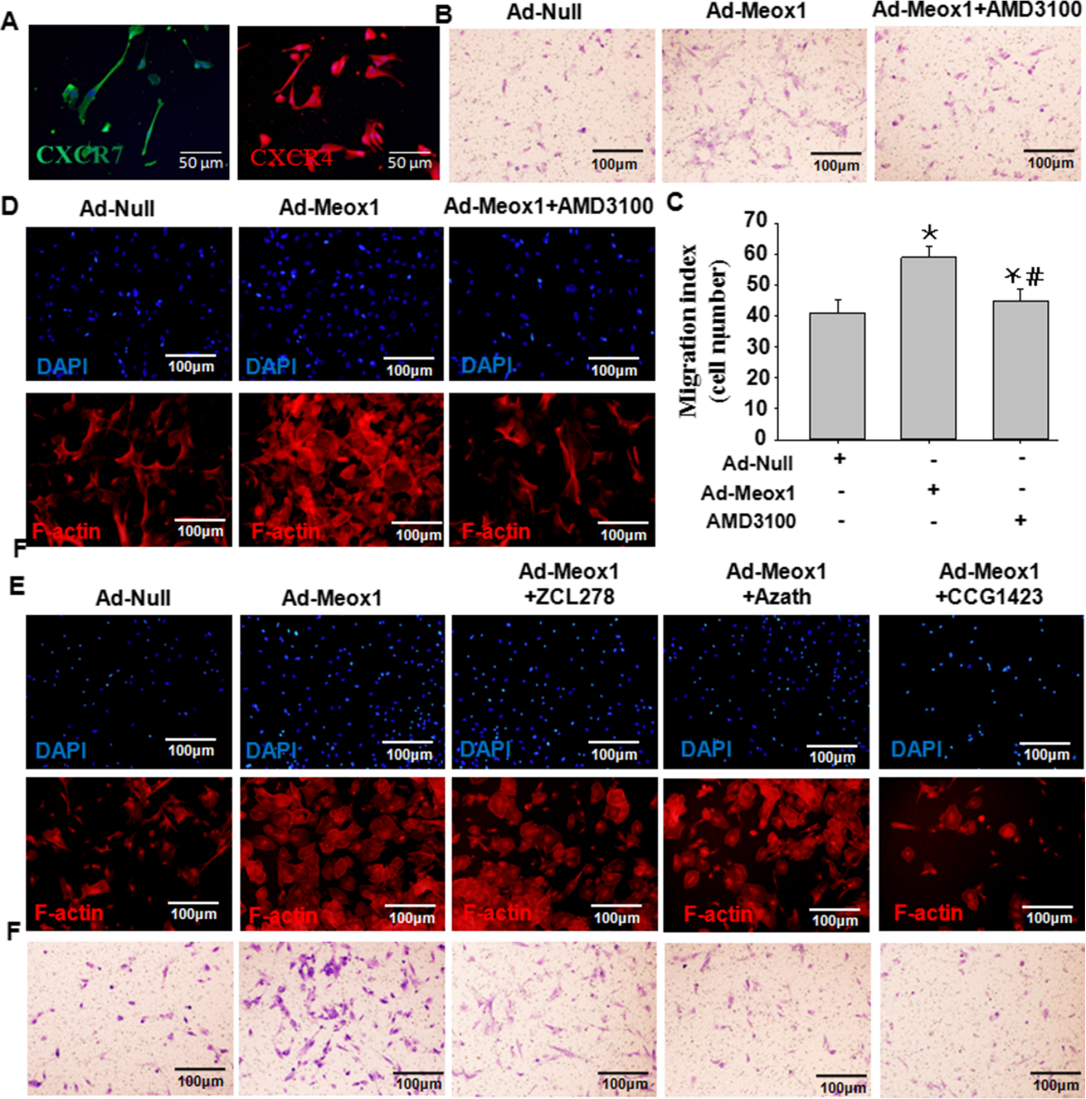
Meox1 triggered Sca-1-positive stem cell migration through RhoA-CDC42-CXCR4 signaling. The experiment was designed to demonstrate the role of RhoA-CDC42-CXCR4 axis in Meox1-mediated adventitia Sca-1-positive stem cell migration during neointimal formation, by observing human umbilical cord Sca-1-positive stem cells (HUCSCs) migration under transwell in vitro. **A** Immunofluorescence staining for CXCR4 and CXCR7. **B** Human umbilical cord Sca-1-positive stem cells (HUCSCs) migration with overexpression of Meox1. **C** Semi-quantitative analysis of HUCSCs migration with treatment of Ad-Meox1 or Ad-shMeox1. Three independent experiments were performed, n = 18, *P < 0.05 vs. HUCSCs treated with ad-Null; ^#^*P* < 0.05 vs. HUCSCs treated with ad-Meox1. **D**, **E** Immunofluorescence staining for F-actin. Ad-Meox1 increased F-actin levels in HUCSCs, the specific effect could be abolished by CXCR4 blocker AMD3100, RhoA inhibitor CCG1423, CDC42 blocker ZCL278, or Rac1 inhibitor Azathioprine, especially in AMD3100 or CCG1423. **F** Meox1-mediated HUCSCs migration with the treatment of RhoA inhibitor CCG1423, CDC42 blocker ZCL278, or Rac1 inhibitor Azathioprine, respectively. Ad-Meox1 increased HUCSCs migration; the specific effect could be abolished by RhoA inhibitor CCG1423, CDC42 blocker ZCL278, or Rac1 inhibitor Azathioprine, especially in Azathioprine

To substantiate the cooperative mechanisms of SDF-1α interaction with CXCR4 in Sca-1+ progenitor cell migration by Meox1, we cultured Sca-1+ progenitor cells from the adventitia layer of human umbilical cords and stained the cells against the CXCR4 antibody (Fig. 6A). Cells were treated either with Ad-Meox1 (CM-AdMeox1) or Ad-Meox1 with AMD3100. H&E staining shows that AMD3100 abrogated the effects observed from Moex1 overexpression. Similarly, Sca-1+ progenitor cell migratory effects that were enhanced by overexpressing Meox1 were abolished by AMD3100 (Fig. 6B, C). Furthermore, the migratory response of Sca-1 progenitor cells to the conditioned medium of VSMC treated with Ad-Meox1 (CM-AdMeox1) was evidently abrogated by CXCR4 blocker AMD3100, indicating that released SDF-1α by Meox1 induced Sca-1+ progenitor cell migration through CXCR4. More importantly, overexpressing-Meox1 in Sca-1 progenitor cells showed an increased migration ability, and the specific effect could be abolished by CXCR4 blocker AMD3100 (Fig. 6B, C).

F-actin has a vital role in mediating cell migration by Rho/CDC42 signaling [24, 25]. Because we have demonstrated the activation of Rho/CDC42 in Sca-1+ progenitor cells by Meox1 (Fig. 3), we aimed to explore the expression of F-actin in CXCR4-inhibited cells. Herein, as shown in Fig. 6D, the overexpression of Meox1 in Sca-1 progenitor cells showed significant expression of F-actin, and AMD3100 could abrogate these effects. Furthermore, inhibitions of CDC42 with its blocker, ZCL278, and inhibition of Rac1 with its inhibitor, Azathioprine, were not able to abolish the enhanced expression of F-actin in Sca-1 progenitor cells induced by overexpression of Meox1. However, the inhibition of RhoA by its inhibitor, CCG1423, could repeal the marked F-actin expression in Meox1 overexpressed cells. Taken together, the process of Meox1-mediating Sca-1+ progenitor cell migration into the neointimal in the injured arteries involved the RhoA-CDC42-CXCR4 signaling pathway.

### CDC42 was involved in SDF-1α expression in VSMC and CXCR4 expression in Sca-1+ stem cells mediated by Meox1

**Fig. 7:**
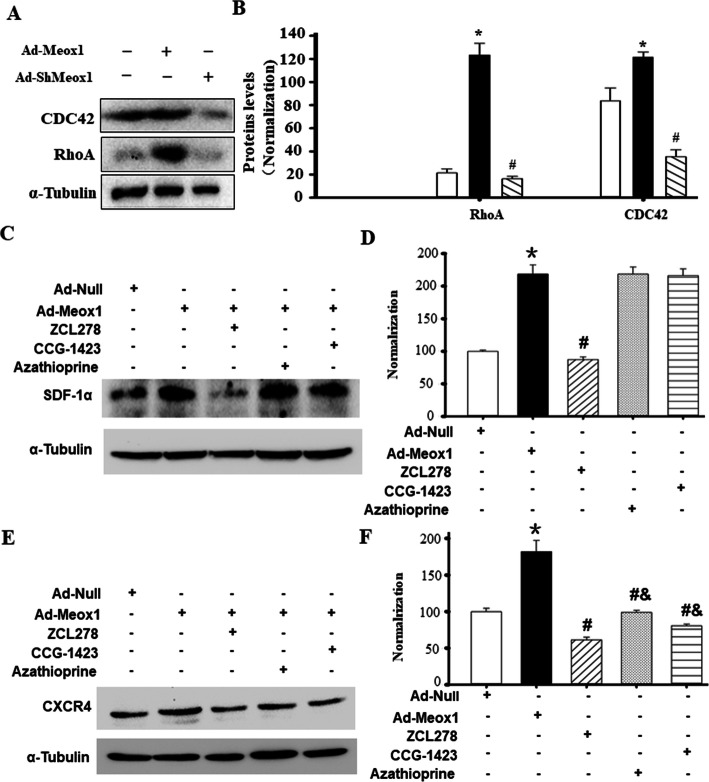
CDC42 was involved in SDF-1α expression in VSMC and CXCR4 expression in Sca-1+ stem cells mediated by Meox1. The aim of the experiment was to explore possible molecular mechanisms of Meox1-induced SDF-1α expression in VSMC and CXCR4 expression in HUCSCs, using RhoA inhibitor CCG1423, CDC42 blocker ZCL278, or Rac1 inhibitor Azathioprine. **A**, **B** Overexpressing Meox1 (Ad-Meox1) increased SDF-1α expressions while knockdown of Meox1 by shRNA (Ad-shMeox1) in VSMCs transfected with treatment of Ad-Meox1 or Ad-shMeox1 for 3 days as determined by western blot (**A**) and semi-quantitative analysis (**B**). Three independent experiments were performed, n = 3, *P < 0.05 vs. VSMC transfected with Ad-Null; ^#^*P* < 0.05 vs. VSMCs transfected with Ad-Meox1. **C**, **D** The SDF-1α expressions in VSMCs transfected with Ad-Meox1, following the addition of RhoA inhibitor CCG1423, CDC42 blocker ZCL278, or Rac1 inhibitor Azathioprine, as determined by western blot (**C**) and semi-quantitative analysis (**D**). Three independent experiments were performed, n = 3, *P < 0.05 vs. VSMCs transfected with Ad-Null; ^#^*P* < 0.05 vs. VSMCs transfected with Ad-Meox1. **E**, **F** CXCR4 expressions in HUCSCs transfected with Ad-Meox1 for 24 h, following the addition of CCG1423, or Azathioprine for 48 h, as determined by western blot (**E**) and semi-quantitative analysis (**F**). Three independent experiments were performed, n = 3, *P < 0.05 vs. HUCSCs treated with Ad-Null; ^#^*P* < 0.05 vs. HUCSCs treated with Ad-Meox1; ^&^*P* < 0.05 vs. Ad-Meox1-transfected HUCSCs treated with CDC42 blocker ZCL278

To better determine the relationship between Meox1 and RhoA-CDC42, overexpression and knockdown of Meox1 were used to treat VSMC in vitro, as shown in Fig. 7A and B, overexpressing Meox1 markedly increased proteins levels of RhoA and CDC42 while knockdown of Meox1 obviously decreased them. Subsequently, to further determine the regulation of SDF-1α by Meox1 in VSMC, we overexpressed VSMC with Meox1 and inhibited these cells with CDC42 (ZCL278), Rac1 (Azathioprine), and RhoA (CCG1423) inhibitors. As shown in Fig. 7C and D, western blot analysis show that the overexpression of Meox1 significantly enhanced SDF-1α protein levels in VSMC. CDC42 blocker, ZCL278, could successfully abolish these significant effects observed with SDF-1α in Moex1 overexpressed cells were. However, inhibiting Rac1 or RhoA did not have any influence on the protein expression level of SDF-1α in Ad-Meox1-treated VSMC. Meanwhile, the CXCR4 protein expression levels were markedly increased by the overexpression of Meox1 in Sca-1+ progenitor cells (Fig. 7E, F). These special effects were markedly abolished by the inhibition of CDC42, Rac1, and RhoA with their respective inhibitors. These results demonstrated that RhoA-CDC42 signaling pathways are involved in Meox1-induced SDF-1α expression in VSMC and CXCR4 expression in Sca-1+ progenitor cells.

## Discussion

In the present study, we made three novel findings. Firstly, Meox1 triggered Sca-1+ progenitor cells within the adventitia wall to migrate into intimal and accelerated neointimal formation. Secondly, SDF-1α-induced by Meox1 in VSMC promoted Sca-1+ progenitor cell migration through activating CXCR4. And lastly, CDC42 was involved in the expression of SDF-1α in VSMC and the expression of CXCR4 in Sca-1+ progenitor cells mediated by Meox1.

Published data have demonstrated that the mobilization and recruitment of ample Sca-1+ progenitor cells present in the adventitia and media of vascular wall are mainly responsible for VSMC accumulation in the intima during vascular remodeling such as intimal hyperplasia and arterial sclerosis [5, 6, 8, 30–32]. In fact, the spatio-temporal distribution of Sca-1-positive progenitor cells in injured blood vessels, at least in part, requires three conditions. First, there should be enough Sca-1+ progenitor cells; second, they should migrate to the injured intima; finally, the cells should acquire VSMC phenotypes to participate in neointima formation. We found that as the Meox1 expression gradually increased within the adventitia, media, and intima, the number of Sca-1-positive progenitor cells also increased, suggesting that Meox1 aided to fulfil the first condition. Furthermore, Sca-1+ positive progenitor cells in injured vessel expressed enhanced expression levels of Rac1, Cdc42, and RhoA, which play a unique role in regulating cytoskeletal proteins-mediated cell motility [26, 27]. More importantly, Meox1-induced SDF-1α in VSMC acts as a signpost to guide the directional migration of these cells into the intimal. These results are consistent with previous studies that found that Meox1 could induce MSC differentiation into VSMC [33]. In a word, the spatio-temporal model of Meox1 expression could trigger the engagement of Sca-1+ progenitor stem cells during the neointima formation.

**Fig. 8:**
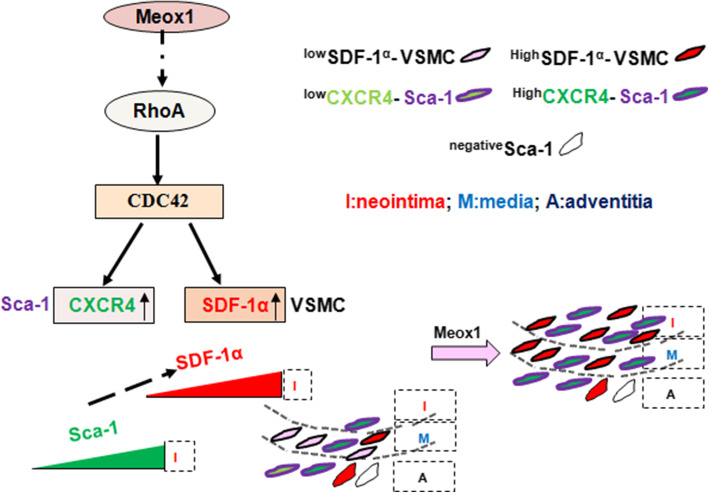
The working model: Spatio-temporal model of Meox1 expression control involvement of Sca-1-positive stem cells in neointima formation through the synergistic effect of Rho/CDC42 and SDF-1α/CXCR4. Meox1 triggered adventitia Sca-1+ progenitor cell migration into neointimal and accelerated neointimal formation. SDF-1α induced by Meox1 in VSMC promoted Sca-1+ progenitor cell migration through activating CXCR4. CDC42 was involved in SDF-1α expression in VSMC and CXCR4 expression in Sca-1+ progenitor cells mediated by Meox1. I, neointima; M, media; A, adventitia

In addition to the Meox1 expression trait seen, our results also show that SDF-1α expressions gradually increased in a time-dependent manner the vascular wall after vascular injury. Subsequently, SDF-1α expressions showed enhanced expression in response to the overexpression of Meox1 in vivo as well as in Ad-Meox1-treated VSMCs in vitro. More importantly, knockdown of Meox1 by Ad-shMeox1 in the injured vessels could significantly reduce SDF-lα expression and decrease neointima formation.

Furthermore, the CDC42 blocker, ZCL278, abolished the Meox1-induced SDF-1α expression in VSMC, contrastingly to the role observed in p38MAPK and NF-κB-induced SDF-1α expression [34, 35]. These results indicate that Meox1 is a potential promoter of SDF-lα via the RhoA-CDC42 signaling pathway. SDF-1α/CXCR4 axis plays a crucial role in neointima formation through activating Sca-1+ progenitor cells [29, 36]. Our results further have shown that the knockdown of Meox1 by shRNA could not only decrease the CXCR4 expression in injured vessels but also significantly diminished the number of Sca-1+ progenitor cells and led to the inhibition of neointima formation. Furthermore, AMD3100, a CXCR4 inhibitor, could also abate the cell numbers, diminish the neointima area in vivo, and partially abrogated cell migration induced by condition medium with Ad-Meox1 in vitro. Moreover, the CDC42 blocker, ZCL278, and the Rac1 inhibitor, Azathioprine, abolished the expression of Meox1-induced CXCR4 in Sca-1+ progenitor cells, which was different from the role of NFAT and ERK1/2 in regulating CXCR4 expression [37]. In summary, these results suggest that Meox1 induces Sca-1+ progenitor cell migration and advance the neointima formation through RhoA-CDC42-CXCR4 signaling mechanisms (Fig. 8).

## Conclusions

Spatio-temporal model of Meox1 expression regulates the involvement of Sca-1+stem cells in the formation of the neointima through the synergistic effect of Rho/CDC42 and SDF-1α/CXCR4 mechanisms.

## Supplementary Information

**Additional file 1: Figure S1.** Traits spatiotemporal expressions of Meox1 and Sca-1+ stem cells during balloon injury-induced neointimal formation. Figure S2. Traits spatiotemporal expressions of Meox1 and Sca-1+ stem cells during balloon injury-induced neointimal formation. FigureS3.Meox1 triggered Sca-1 positive stem cells migration through RhoA-CDC42-CXCR4 signaling.

## Data Availability

Please contact corresponding author for data requests.

## Abbreviations

α-SMA: Alpha smooth muscle actin
CXCR4: C-X-C motif chemokine receptor 4
DAPI: 4′,6-Diamidino-2-phenylindole
ERK1/2: Extracellular signal-regulated kinase1/2
MAPK: Mitogen-activated protein kinase
NF-κB: Nuclear Factor Kappa B
M: Mol/L
PCI: Percutaneous coronary intervention
PBS: Phosphate-buffered saline
PVDF: Polyvinylidene fluoride
HRP: Horseradish peroxidase
IF: Immunofluorescence
HUCSCs: Human umbilical cord Sca-1-positive stem cells
Sca-1: Stem cell antigen-1
SDF-1α: Stromal cell derived factor 1 alpha
VSMC: Vascular smooth muscle cell
