# Supplementary material for: Spatio-temporal model of Meox1 expression control involvement of Sca-1-positive stem cells in neointima formation through the synergistic effect of Rho/CDC42 and SDF-1α/CXCR4
Source: Stem Cell Res Ther. 2021 Jul 7;12:387. doi: 10.1186/s13287-021-02466-8 (PMC8262022; doi:10.1186/s13287-021-02466-8)
Supplement: Supplementary file 1 — Additional file 1: Figure S1. Traits spatiotemporal expressions of Meox1 and Sca-1+ stem cells during balloon injury-induced neointimal formation. Figure S2. Traits spatiotemporal expressions of Meox1 and Sca-1+ stem cells during balloon injury-induced neointimal formation. FigureS3.Meox1 triggered Sca-1 positive stem cells migration through RhoA-CDC42-CXCR4 signaling. [file 13287_2021_2466_MOESM1_ESM.docx]

**Spatio-temporal model of Meox1 expression control involvement of Sca-1 positive stem cells in neointima formation through synergistic effect of Rho/CDC42 and SDF-1ɑ/CXCR4**

Yan Wu^1^*, Yuan-jin Li^2^*,Liu-liu Shi^1^*, Yun Liu^1^, Yan Wang^1^, Xin Bao^1^, Wei Xu^1^, Lu-yuan Yao^1^, MagdaleenaNaemi Mbadhi^1^, Long Chen^3^, Shan Li^4^, Xing-yuan Li^1^, Zhi-feng Zhang^1,5^, Sen Zhao^1^, Ruo-lan Zhang^1^, Shi-You Chen^6^, Jing-xuan Zhang^1,5#^,Jun-mingTang^1,5#^

^1^Department of Physiology, Hubei Key Laboratory of Embryonic Stem Cell Research, Faculty of Basic Medical Sciences, Hubei University of Medicine, Shiyan, Hubei 442000, PR China

^2^Hebei University of Medicine, Hubei 442000, China.

^3^Cental Lab, Guoyao-Dongfeng Hospital, Hubei University of Medicine, Hubei, 442000, China.

^4^Department of Biochemistry, Faculty of Basic Medical Sciences, Hubei University of Medicine, Shiyan, Hubei 442000, PR China

^5^Institute of Basic Medical Sciences, Institute of Biomedicine, Hubei University of Medicine, Hubei442000, China.

^6^The Department of Surgery, University of Missouri, Columbia, U.S.A.

*Co-fisrt author :Yan Wu, Yuan-jin Li&Liu-liu Shi

^#^Co-corresponding author: Jing-xuan Zhang &Jun-Ming Tang, MD, PhD

Tel.:+86-719/8637706; Fax: 86-719/8637792;

E-mail: tangjm416@163.com

**SFigure.1. Traits spatiotemporal expressions of Meox1 and Sca-1+ stem cells during balloon injury-induced neointimal formation**

**
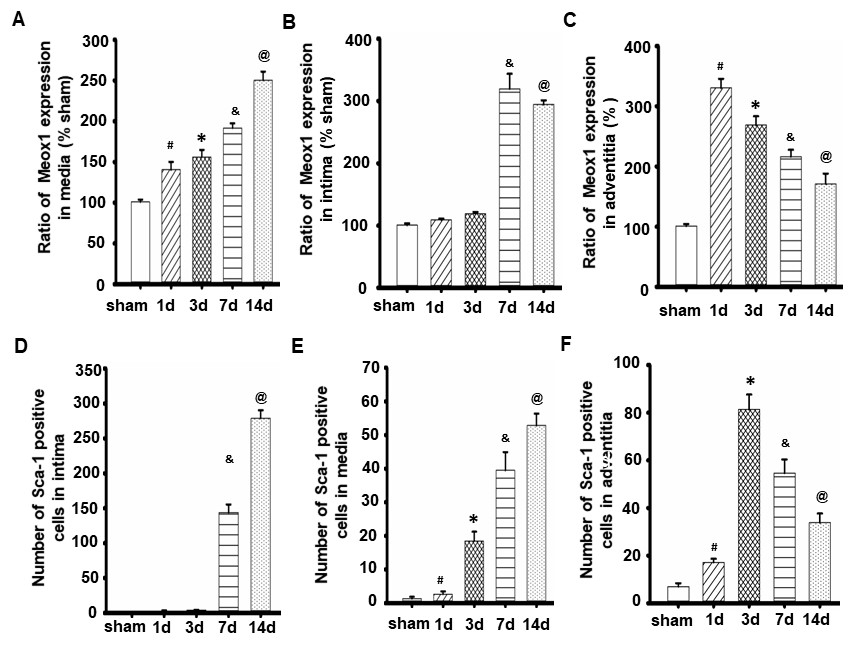
**

(A-F)Rats were suffered from carotid artery balloon injury. Representative sections of sham-operation and injury arteries at the indicated time were stained with Meox1 or Sca-1. (A-C)Semi-quantitative analysis of optical density value of immunohistochemical staining of Meox1 were determined within media or intima of sham-operation and injury arteries by image-Pro Plus software, percentage of Meox1 expression at adventitia, media or intima in the indicated time compared to sham group were calculated. (D-F) Number of Sca-1+ progenitor cells at adventitia , media or intimawere determined in the indicated time compared to sham group at adventitia of vessel wall by image-Pro Plus software. n=6, ^#^*P*<0.05 compared to sham group; ^*^*P*<0.05 vs. 1st day after injury; ^&^*P*<0.05 vs. 2nd day after injury; ^@^*P*<0.05 vs. 7th day after injury.

**sFigure2.Meox1 induce Sca-1^+^ stem cells migration into neointima through activating SDF-1 ɑ signaling in ɑ-SMA+ cells.**

Rats were suffered from carotid artery balloon injury. SDF-1αexpressions were analyzed in VSMC transfected with treatment of Ad-shMeox1 for 14 days. Semi-quantitative analysis ofSDF-1α in VSMC of figure4E. n=3, ^&^*P*<0.05 vs. Ad-Null.

**sFigure3. Meox1 triggered Sca-1 positive stem cells migration through RhoA-CDC42-CXCR4 signaling**

Meox1-mediated Sca-1 positive stem cells migrationwere analyzed in transwell system with the treatment of RhoA inhibitor CCG1423 (10 μM), CDC42 blocker ZCL278 (10 μM) or Rac1 inhibitor Azathioprine(1 μM), respectively.n=6, ^*^*P*<0.05 vs. Ad-Null;^#^P<0.05 vs. Ad-Meox1.
